# Adenovirus Carrying Gene Encoding *Haliotis discus discus* Sialic Acid Binding Lectin Induces Cancer Cell Apoptosis

**DOI:** 10.3390/md12073994

**Published:** 2014-06-30

**Authors:** Xinyan Yang, Liqin Wu, Xuemei Duan, Lianzhen Cui, Jingjing Luo, Gongchu Li

**Affiliations:** College of Life Sciences, Zhejiang Sci-Tech University, Hangzhou 310018, Zhejiang, China; E-Mails: 499104236@qq.com (X.Y.); 2529594272@qq.com (L.W.); 287722892@qq.com (X.D.); 819517567@qq.com (L.C.); deepstoh@163.com (J.L.)

**Keywords:** *Haliotis discus discus* sialic acid binding lectin, adenoviruses, apoptosis, Bcl-2

## Abstract

Lectins exist widely in marine bioresources such as bacteria, algae, invertebrate animals and fishes. Some purified marine lectins have been found to elicit cytotoxicity to cancer cells. However, there are few reports describing the cytotoxic effect of marine lectins on cancer cells through virus-mediated gene delivery. We show here that a replication-deficient adenovirus-carrying gene encoding *Haliotis discus discus* sialic acid binding lectin (Ad.FLAG-HddSBL) suppressed cancer cell proliferation by inducing apoptosis, as compared to the control virus Ad.FLAG. A down-regulated level of anti-apoptosis factor Bcl-2 was suggested to be responsible for the apoptosis induced by Ad.FLAG-HddSBL infection. Further subcellular localization studies revealed that HddSBL distributed in cell membrane, ER, and the nucleus, but not in mitochondria and Golgi apparatus. In contrast, a previously reported mannose-binding lectin *Pinellia pedatisecta* agglutinin entered the nucleus as well, but did not distribute in inner membrane systems, suggesting differed intracellular sialylation and mannosylation, which may provide different targets for lectin binding. Further cancer-specific controlling of HddSBL expression and animal studies may help to provide insights into a novel way of anti-cancer marine lectin gene therapy. Lectins may provide a reservoir of anti-cancer genes.

## 1. Introduction

Lectins are carbohydrate-binding proteins which have become useful tools for recognizing a variety of cell types due to their carbohydrate recognition specificity [1,2]. Some lectins such as *Maackia amurensis* seed lectin [3], Concanavalin A [4], and *Polygonatum cyrtonema* lectin [5] elicit anticancer effect by inducing apoptosis or autophagy, whereas a mannose-binding plant lectin *Pinellia pedatisecta* agglutinin (PPA) preferentially recognizes drug resistant K562/ADR leukemia cells through binding with sarcolemmal membrane associated protein, and enhances the phagocytosis of K562/ADR by macrophages [6]. Furthermore, through gene delivery, PPA induces cancer cell death through interacting with the methylosome that contains protein arginine methyltransferase 5 and methylosome protein 50 [7].

Sialic acid-binding lectins (SBLs), widely exist in plant and animals, and comprise a large family of lectins showing sialic acid binding activity and various biological functions. To date, most studies of SBLs were performed in animal adhesion molecules Siglecs [8], which are expressed on the cell membrane of various immune cells including macrophages [9,10], B cells [11], neutrophils [12], as well as some cancer cells [13,14]. Siglecs regulate various immune responses. For example, Siglec-1 is a macrophage restricted cell surface receptor and contributes to sialylated pathogen uptake, antigen presentation, and cytokine production [15]. Siglec-E selectively regulates neutrophil recruitment during acute inflammation [12]. CD22 (Siglec-2) is expressed on B cells and dendritic cells, and regulates B cell receptor signaling, cell survival, proliferation, antibody production, as well as CD8+ T cell regulation [11,16]. The CD33-related Siglecs are expressed primarily on leukocytes in a cell type-specific manner and are involved in modulation of inflammatory and immune responses [17]. Meanwhile, Siglecs such as CD22 and CD33 expressed on malignant cells have become therapeutic targets for a variety of cancer types, such as lung cancer [13] and leukemia [14,18,19,20]. In addition to Siglecs, some other SBLs from plant and animals have been purified and characterized, including SBLs isolated from *Polygonatum cyrtonema* [21], frogs *Rana catesbeiana* and *Rana japonica* oocytes [22], razor clam *Solen grandis* [23], and manila clam *Venerupis philippinarum* [24]. Anticancer effects have been suggested for some of these SBLs, such as egg lectins from frogs *Rana catesbeiana* and *Rana japonica* [22,25,26], as well as *Polygonatum cyrtonema* lectin [5,27].

Gene delivery of exogenous lectins through viral vectors may provide an alternative way to induce cancer cell death [7]. However, at the present, there are few reports describing the cytotoxic effect of marine lectins on cancer cells through virus-mediated gene delivery. In this work, a gene encoding *Haliotis discus discus* sialic acid binding lectin (HddSBL) was genetically inserted into an adenoviral vector to form a recombinant adenovirus Ad.FLAG-HddSBL. The cytotoxicity of Ad.FLAG-HddSBL on cancer cells was analyzed. Furthermore, to analyze the underlying mechanism of the HddSBL induced cytotoxicity, the subcellular distribution of HddSBL in cancer cells was investigated through transfection of cells with plasmid pEGFP-HddSBL-C1, followed by observation under a confocal laser scanning microscope.

## 2. Results and Discussion

**Figure 1:**
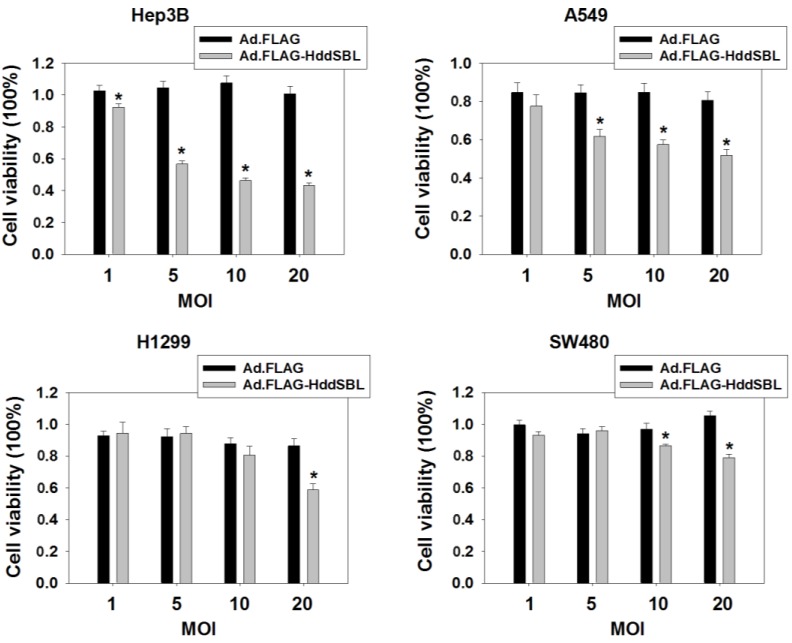
Ad.FLAG-HddSBL suppressed cancer cell proliferation. Hepatocellular carcinoma cell lines Hep3B, lung cancer cell line A549 and H1299, as well as colorectal cancer cell line SW480 were treated with Ad.FLAG, Ad.FLAG-HddSBL at 1, 5, 10, or 20 multiplicity of infections (MOIs) for 96 h. Cell viability was analyzed through MTT assay. Values from at least six repeats were calculated as percent of PBS control and presented as mean ± SEM. * *p* < 0.05.

To evaluate the cytotoxic effect of exogenous HddSBL, cancer cells including hepatocellular carcinoma cell line Hep3B, lung cancer cell lines A549 and H1299, as well as colorectal carcinoma cell line SW480 were treated with Ad.FLAG-HddSBL or the control adenovirus Ad.FLAG. As compared to Ad.FLAG, Ad.FLAG-HddSBL significantly suppressed the *in vitro* proliferation of these cancer cells, as determined by MTT assay (Figure 1). The suppressive effect of Ad.FLAG-HddSBL on cancer cells took place in a dose- and cell line-dependent manner. Data indicated the cytotoxicity of exogenous HddSBL on various cancer cells, and the differential effect of HddSBL may be due to varied forms of intracellular sialic acids modification.

**Figure 2:**
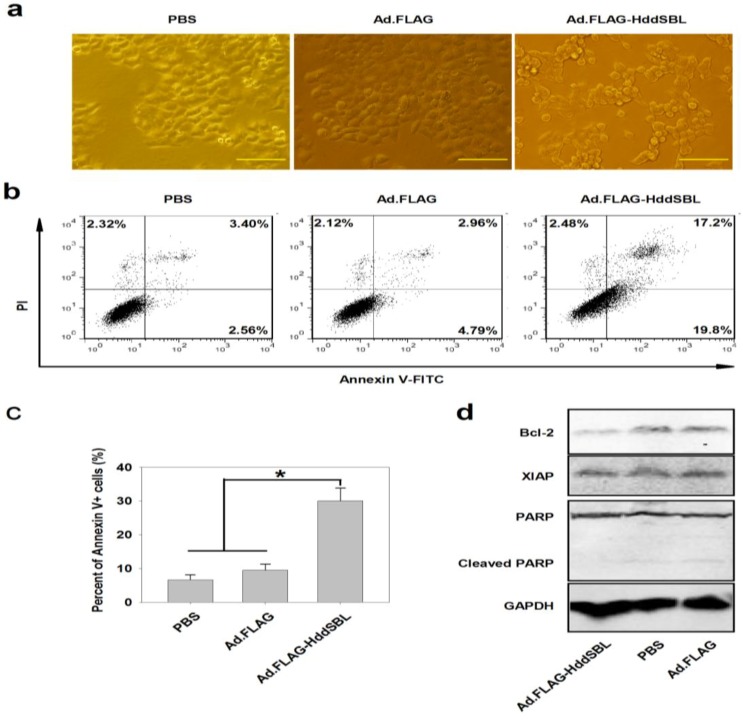
Ad.FLAG-HddSBL induced apoptosis in Hep3B cells. Hep3B cells treated with Ad.FLAG or Ad.FLAG-HddSBL at 20 MOI as well as PBS control for 48 h. (**a**) Morphology of apoptosis induced by Ad.FLAG-HddSBL (bars: 200 μm); (**b**) Cells were stained with Annexin V-FITC and PI followed by analysis under a flow cytometer; (**c**) The percent of Annexin V-positive cells from three repeats were shown as mean ± SEM (* *p* < 0.05); (**d**) Cell lysates were analyzed by Western blot for levels of Bcl-2, XIAP, and PARP. GAPDH served as the loading control.

To investigate the mechanism of HddSBL induced cytotoxicity, hepatocellular carcinoma cell line Hep3B was treated with PBS, Ad.FLAG, or Ad.FLAG-HddSBL, followed by observation under a microscope, as well as staining with Annexin V-FITC and propidium iodide (PI), a common method for apoptotic cell staining, and analyzing under a flow cytometer. Typical morphological change resulting from apoptosis was observed in cells treated with Ad.FLAG-HddSBL (Figure 2a). Figure 2b showed that Ad.FLAG-HddSBL induced a higher percent of Annexin V^+^/PI^−^ and Annexin V^+^/PI^+^ cells, as compared to the cells treated with either PBS or Ad.FLAG. Significant differences achieved from three repeats were shown in Figure 2c. Results indicate that exogenous expression of HddSBL induced apoptosis in Hep3B liver cancer cells.

To analyze the underlying mechanism of apoptosis induced by Ad.FLAG-HddSBL, Hep3B cells treated with Ad.FLAG-HddSBL, Ad.FLAG, or PBS were lysed, and apoptotic signaling elements were investigated by Western blot. As shown in Figure 2d, compared to PBS and Ad.FLAG controls, Ad.FLAG-HddSBL even prohibited the cleavage of poly (ADP-ribose) polymerase (PARP), a substrate for various caspases, suggesting that Ad.FLAG-HddSBL possibly did not induce apoptosis through activating caspases. However, Ad.FLAG-HddSBL significantly suppressed levels of anti-apoptosis factor Bcl-2. The densitometry analysis showed that the band density of Bcl-2 adjusted by GAPDH were 0.7 ± 0.09, 0.91 ± 0.05, and 0.87 ± 0.06 for Ad.FLAG-HddSBL, PBS, and Ad.FLAG, respectively (Ad.FLAG-HddSBL *vs.* PBS or Ad.FLAG: *p* < 0.05). Another anti-apoptosis element X-linked inhibitor of apoptosis protein (XIAP) was not apparently altered by Ad.FLAG-HddSBL. Data suggested that exogenous expression of HddSBL induced apoptosis in Hep3B cells through down-regulating anti-apoptosis factor Bcl-2.

**Figure 3:**
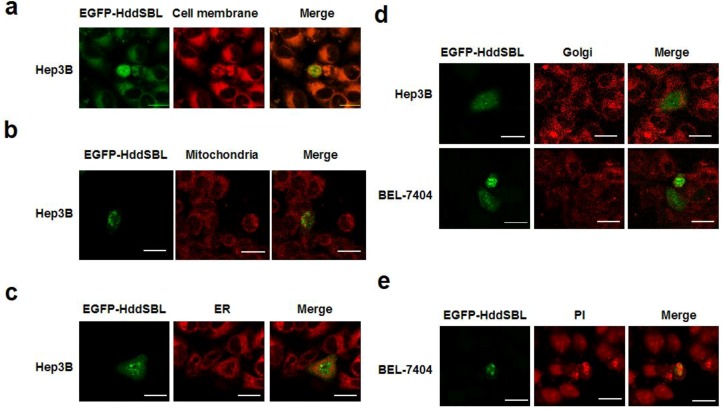
Subcellular distribution of HddSBL. Hep3B or BEL-7404 cells were transfected with pEGFP-HddSBL-C1. Cells were then stained with DiI (**a**); Mito Tracker Red Mitochondrion-Selective Probe (**b**); Golgi-Tracker Red (**c**); ER-Tracker Red (**d**); and PI (**e**); followed by analysis under a confocal laser scanning microscope. Bars show 20 μm. The diagrams are representative of at least four repeats.

Intracellular distribution of HddSBL was investigated to further study the underlying mechanism of the HddSBL induced apoptosis. Liver cancer cells Hep3B or BEL-7404 were transfected with plasmids pEGFP-HddSBL-C1, followed by subcellular staining and observation under a confocal laser scanning microscope. As shown in Figure 3a–d, distribution of HddSBL was observed in the cell membrane and ER, but not in the mitichondria and Golgi apparatus. Interestingly, at a late stage, HddSBL entered the PI high staining area (Figure 3e), suggesting the entrance of HddSBL into the nucleus.

**Figure 4:**
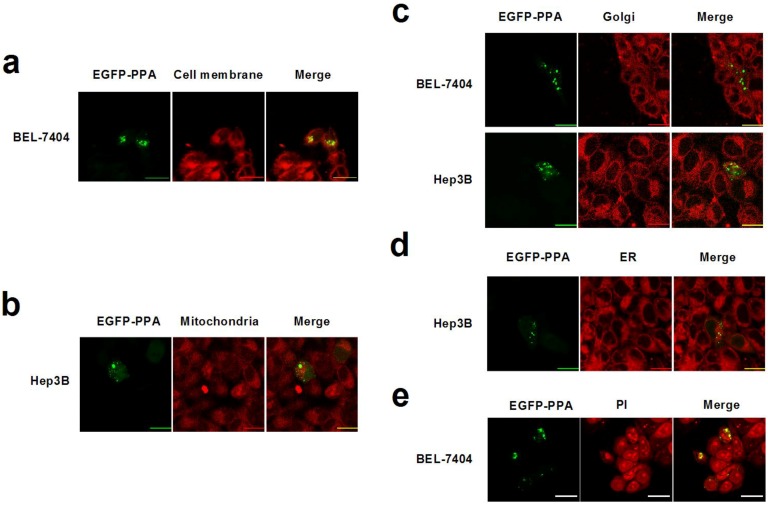
Subcellular distribution of PPA. Hep3B or BEL-7404 cells were transfected with pEGFP-HddSBL-C1. Cells were then stained with DiI (**a**); Mito Tracker Red Mitochondrion-Selective Probe (**b**); Golgi-Tracker Red (**c**); ER-Tracker Red (**d**); and PI (**e**); followed by analysis under a confocal laser scanning microscope. Bars show 20 μm. The diagrams are representative of at least four repeats.

We previously determined that mannose-binding lectin PPA also entered the nucleus and induced cell death [7]. Therefore, plasmid pEGFP-PPA-C1 was transfected into Hep3B cells, and the distribution of PPA was examined as well. As shown in Figure 4a–d, although slight location of PPA on the cell membrane was detected, the majority of PPA did not distribute in the inner membrane systems, including mitochondria, ER, and Golgi apparatus. The entrance of PPA into the nucleus was verified by analysis under a confocal laser scanning microscope (Figure 4e). The different distribution of HddSBL and PPA suggested their different interactions with intracellular molecules such as carbohydrates and proteins.

The subcellular localization studies showed that HddSBL and PPA distributed differently in cell membrane and cytoplasm. Glycans are linked and modified to proteins and lipids by glycosyltransferases and glycosidases in secretory pathways, which play important roles in regulating cell adhesion, molecular trafficking, and signal transduction [28]. Since lectins are carbohydrate-specific proteins, the varied distribution of these two lectins may suggest different sialylation and mannosylation patterns on subcellular organelles. The distribution of HddSBL may reflect high levels of terminal sialic acids on the cell membrane and ER, as well as the nucleus, whereas the PPA collocalization with DNA may reflect a low level of terminal mannose in cytoplasm but a high level of terminal mannose in the nucleus.

Sialic acid is a nine-carbon sugar, naturally occurring as the terminal carbohydrate residues, involved in various biological functions. Many studies have demonstrated that cell surface sialic acids play an important role for the attachment and infection of viruses [29,30,31,32,33]. However, sialic acids were also found in regulating various cellular functions. For example, cell surface α2,3- and α2,6-sialic acid determinants were correlated with pancreatic carcinoma cell migration [34]. The terminal sialic acids decreased on the cell surface of apoptotic lymphocytes, and induced a signal for macrophage phagocytosis [35]. The absence of sialylation and exposure of galactose residues on the surface of platelets resulted in recognition and clearance by macrophages and hepatocytes [36]. In addition to the role of recognition molecules, sialic acids also acted as an intracellular signaling molecule regulating cell proliferation and differentiation [37]. Therefore, intracellular expression of sialic acid-binding lectins may interfere with many cell membrane and cytoplasmic signaling molecules, leading to diversified biological outcomes. In our data, exogenous HddSBL was found to be distributed in the cell membrane, ER, and the nucleus. The binding of HddSBL to carbohydrate chains with sialic acid terminal residues in these sites may interfere with various signaling pathways, as well as glaycosylation processes. Previously, a binding of intracellular polysialic acid antigens by a monoclonal antibody induced apoptosis in human cancer cell lines [38]. We further determined that the exogenous expression of a sialic acid-binding lectin HddSBL down-regulated anti-apoptotic factor Bcl-2 and led to cell apoptosis. These studies demonstrated that not only that sialic acids distributed on the extracellular domain of cell membrane receptors such as podoplanin [3], but also that intracellular sialic acids could provide therapeutic targets for various cancer cells.

## 3. Experimental Section

### 3.1. Cell Culture and Transfection

Heptocellular carcinoma cell lines Hep3B and BEL-7404, lung cancer cell lines A549 and H1299, as well as colorectal carcinoma cell line SW480 were obtained from American Type Culture Collection (Rockville, MD, USA). Cells were maintained in Dulbecco’s modified Eagle’s medium supplemented with 10% fetal bovine serum, 1% penicillin/streptomycin solution, and 1% l-Glutamine. Appropriate amounts of plasmids were transfected into cells by using Thermo Scientific TurboFect Transfection Reagent (Thermo Fisher Scientific Inc., Waltham, MA, USA) following the manufacturer’s instruction.

### 3.2. Plasmid Construction

The plasmid pGH-genes carrying DNA sequences encoding FLAG tagged *Haliotis discus discus* sialic acid binding lectin (HddSBL, GenBank accession no. EF103404) were purchased from Shanghai Generay Biotech Co., Ltd, Shanghai, China. The sequences were cut with *Xho*I, and then inserted into the corresponding site of pEGFP-C1 to form plasmids pEGFP-HddSBL-C1.

### 3.3. Adenoviral Construction

The FLAG tagged sequence of HddSBL was amplified from pGH-HddSBL through polymerase chain reaction. The products were inserted into pCA13 to form pCA13-FLAG-HddSBL. The expression cassette of FLAG-HddSBL was digested from plasmids pCA13-FLAG-HddSBL with *Bgl*II and inserted into the corresponding site of plasmid pShuttle, forming plasmids pShuttle-FLAG-HddSBL, which were subsequently transformed into strain BJ5183. Viral genome of Ad.FLAG-HddSBL was generated through homologous recombination between pShuttle-FLAG-HddSBL and viral skeleton plasmid pAdEasy-1, followed by transfection into 293 A cells after linearized by *Pac*I. Adenoviruse Ad.FLAG-HddSBL was then produced and virus titers were determined by Titer-EZ adenoviral titer detection Reagent (Shang Hai SBO Medical Biotechnology Co., Ltd, Shanghai, China) following the manufacturer’s instruction.

### 3.4. Subcellular Staining and Colocalization Study

ER-Tracker Red, Golgi-Tracker Red, propidium iodide (PI), and DiI were purchased from Beyotime Institute of Biotechnology (Shanghai, China). Mito Tracker Red Mitochondrion-Selective Probe was purchased from Invitrogen (Grand Island, NY, USA). Hep3B or BEL-7404 Cells were transfected with pEGFP-HddSBL-C1 or pEGFP-PPA-C1 for 24 h or 48 h, followed by staining with ER-Tracker Red, Golgi-Tracker Red, Mito Tracker Mitochondrion Selective Probes, PI, or DiI, and observed under a confocal laser scanning microscope (Nikon, Inc., Tokyo, Japan).

### 3.5. Cytotoxicity Detection and Flow Cytometry Assay

Cells were plated on 96-well plates at 5 × 10^3^ per well one day before infected with adenoviruses. Then cells were infected with adenoviruses at corresponding multiplicity of infections (MOI) for 96 h. The cytotoxicity was examined by 3-(4,5-dimethylthiazol-2-yl)-2,5-diphenyltetrazolium bromide (MTT) assays. Meanwhile, cells were infected with adenoviruses at 20MOI for 48 h. Cells were then collected and stained with Annexin V-FITC Apoptosis Detection Kit (KeyGEN Biotech Co., Ltd., Nanjing, China) following the manufacturer’s instruction, and analyzed under a BD FACSAria flow cytometry (BD Biosciences, San Jose, CA, USA).

### 3.6. Western Blotting Analysis

The cell extracts were subjected to SDS-PAGE and electroblotted onto nitrocellulose membranes. The membranes were then blocked with Tris-buffered saline and Tween 20 contaning 5% of bovine serum albumin at room temperature for 2 h and incubated with corresponding antibodies overnight at 4 °C. The membranes were washed and incubated with appropriate dilution of IRDye 800 donkey anti-mouse IgG or IRDye 700 donkey anti-rabbit IgG (LI-COR, Inc., Lincoln, NA, USA) for 1 h at room temperature. After washing with Tris-buffered saline, the membranes were then analyzed by an Odyssey Infrared Imaging System (LI-COR, Inc.).

The rabbit anti-PARP antibody was purchased from Santa Cruz biotechnology Inc. (Santa Cruz, CA, USA). Rabbit anti-XIAP antibody was purchased from Epitomics (Burlingame, CA, USA). Rabbit anti-GAPDH antibody and rabbit anti-Bcl-2 antibody were purchased from Cell Signaling Technology Inc. (Danvers, MA, USA).

## 4. Conclusions

We show here that adenovirus-carrying gene encoding HddSBL induced apoptosis and suppressed *in vitro* proliferation of various cancer cells. The down-regulation of anti-apoptosis factor Bcl-2 may be responsible for the HddSBL induced apoptosis. Further cancer-specific controlling of HddSBL expression and animal studies may help to provide insights into a novel way of anti-cancer lectin gene therapy. Lectins widely existed in the marine bioresources may provide a huge reservoir of genes for cancer gene therapies.

## References

[1] Sharon N., Lis H. (1989). Lectins as cell recognition molecules. Science.

[2] Sharon N. (2007). Lectins: Carbohydrate-specific reagents and biological recognition molecules. J. Biol. Chem..

[3] Ochoa-Alvarez J.A., Krishnan H., Shen Y., Acharya N.K., Han M., McNulty D.E., Hasegawa H., Hyodo T., Senga T., Geng J.G. (2012). Plant lectin can target receptors containing sialic acid, exemplified by podoplanin, to inhibit transformed cell growth and migration. PLoS One.

[4] Liu B., Min M.W., Bao J.K. (2009). Induction of apoptosis by concanavalin a and its molecular mechanisms in cancer cells. Autophagy.

[5] Liu B., Cheng Y., Bian H.J., Bao J.K. (2009). Molecular mechanisms of polygonatum cyrtonema lectin-induced apoptosis and autophagy in cancer cells. Autophagy.

[6] Chen K., Yang X., Wu L., Yu M., Li X., Li N., Wang S., Li G. (2013). *Pinellia pedatisecta* agglutinin targets drug resistant K562/ADR leukemia cells through binding with sarcolemmal membrane associated protein and enhancing macrophage phagocytosis. PLoS One.

[7] Lu Q., Li N., Luo J., Yu M., Huang Y., Wu X., Wu H., Liu X.Y., Li G. (2012). *Pinellia pedatisecta* agglutinin interacts with the methylosome and induces cancer cell death. Oncogenesis.

[8] Jandus C., Simon H.U., von Gunten S. (2011). Targeting siglecs—A novel pharmacological strategy for immuno- and glycotherapy. Biochem. Pharmacol..

[9] O’Neill A.S., van den Berg T.K., Mullen G.E. (2013). Sialoadhesin—A macrophage-restricted marker of immunoregulation and inflammation. Immunology.

[10] Kopatz J., Beutner C., Welle K., Bodea L.G., Reinhardt J., Claude J., Linnartz-Gerlach B., Neumann H. (2013). Siglec-h on activated microglia for recognition and engulfment of glioma cells. Glia.

[11] Jellusova J., Nitschke L. (2011). Regulation of B cell functions by the sialic acid-binding receptors Siglec-G and CD22. Front Immunol..

[12] McMillan S.J., Sharma R.S., McKenzie E.J., Richards H.E., Zhang J., Prescott A., Crocker P.R. (2013). Siglec-E is a negative regulator of acute pulmonary neutrophil inflammation and suppresses cd11b beta2-integrin-dependent signaling. Blood.

[13] Tuscano J.M., Kato J., Pearson D., Xiong C., Newell L., Ma Y., Gandara D.R., O’Donnell R.T. (2012). CD22 antigen is broadly expressed on lung cancer cells and is a target for antibody-based therapy. Cancer Res..

[14] Hoelzer D. (2012). Anti-CD22 therapy in acute lymphoblastic leukaemia. Lancet Oncol..

[15] Klaas M., Oetke C., Lewis L.E., Erwig L.P., Heikema A.P., Easton A., Willison H.J., Crocker P.R. (2012). Sialoadhesin promotes rapid proinflammatory and type I Ifn responses to a sialylated pathogen, *Campylobacter jejuni*. J. Immunol..

[16] Ma D.Y., Suthar M.S., Kasahara S., Gale M., Clark E.A. (2013). CD22 is required for protection against west nile virus infection. J. Virol..

[17] Crocker P.R., McMillan S.J., Richards H.E. (2012). CD33-related siglecs as potential modulators of inflammatory responses. Ann. N. Y. Acad. Sci..

[18] Haso W., Lee D.W., Shah N.N., Stetler-Stevenson M., Yuan C.M., Pastan I.H., Dimitrov D.S., Morgan R.A., FitzGerald D.J., Barrett D.M. (2013). Anti-CD22-chimeric antigen receptors targeting B-cell precursor acute lymphoblastic leukemia. Blood.

[19] Schweizer A., Wohner M., Prescher H., Brossmer R., Nitschke L. (2012). Targeting of CD22-positive B-cell lymphoma cells by synthetic divalent sialic acid analogues. Eur. J. Immunol..

[20] Herrmann H., Cerny-Reiterer S., Gleixner K.V., Blatt K., Herndlhofer S., Rabitsch W., Jager E., Mitterbauer-Hohendanner G., Streubel B., Selzer E. (2012). CD34(+)/CD38(−) stem cells in chronic myeloid leukemia express Siglec-3 (CD33) and are responsive to the CD33-targeting drug gemtuzumab/ozogamicin. Haematologica.

[21] Liu B., Wu J.M., Li J., Liu J.J., Li W.W., Li C.Y., Xu H.L., Bao J.K. (2010). Polygonatum cyrtonema lectin induces murine fibrosarcoma l929 cell apoptosis and autophagy via blocking ras-raf and pi3k-akt signaling pathways. Biochimie.

[22] Lu C.X., Nan K.J., Lei Y. (2008). Agents from amphibians with anticancer properties. Anticancer Drugs.

[23] Yang J., Wei X., Liu X., Xu J., Yang D., Fang J., Hu X. (2012). Cloning and transcriptional analysis of two sialic acid-binding lectins (SABLs) from razor clam *Solen grandis*. Fish Shellfish Immunol..

[24] Li C., Yu S., Zhao J., Su X., Li T. (2011). Cloning and characterization of a sialic acid binding lectins (SABL) from manila clam *Venerupis philippinarum*. Fish Shellfish Immunol..

[25] Tatsuta T., Hosono M., Sugawara S., Kariya Y., Ogawa Y., Hakomori S., Nitta K. (2013). Sialic acid-binding lectin (leczyme) induces caspase-dependent apoptosis-mediated mitochondrial perturbation in jurkat cells. Int. J. Oncol..

[26] Tatsuta T., Hosono M., Miura Y., Sugawara S., Kariya Y., Hakomori S., Nitta K. (2013). Involvement of ER stress in apoptosis induced by sialic acid-binding lectin (leczyme) from bullfrog eggs. Int. J. Oncol..

[27] Wang S.Y., Yu Q.J., Bao J.K., Liu B. (2011). Polygonatum cyrtonema lectin, a potential antineoplastic drug targeting programmed cell death pathways. Biochem. Biophys. Res. Commun..

[28] Ohtsubo K., Marth J.D. (2006). Glycosylation in cellular mechanisms of health and disease. Cell.

[29] Mahon P.J., Mirza A.M., Iorio R.M. (2011). Role of the two sialic acid binding sites on the newcastle disease virus HN protein in triggering the interaction with the F protein required for the promotion of fusion. J. Virol..

[30] Mistry N., Inoue H., Jamshidi F., Storm R.J., Oberste M.S., Arnberg N. (2011). Coxsackievirus A24 variant uses sialic acid-containing o-linked glycoconjugates as cellular receptors on human ocular cells. J. Virol..

[31] Oshansky C.M., Pickens J.A., Bradley K.C., Jones L.P., Saavedra-Ebner G.M., Barber J.P., Crabtree J.M., Steinhauer D.A., Tompkins S.M., Tripp R.A. (2011). Avian influenza viruses infect primary human bronchial epithelial cells unconstrained by sialic acid alpha2,3 residues. PLoS One.

[32] Leung H.S., Li O.T., Chan R.W., Chan M.C., Nicholls J.M., Poon L.L. (2012). Entry of influenza a virus with a alpha2,6-linked sialic acid binding preference requires host fibronectin. J. Virol..

[33] Neu U., Hengel H., Blaum B.S., Schowalter R.M., Macejak D., Gilbert M., Wakarchuk W.W., Imamura A., Ando H., Kiso M. (2012). Structures of merkel cell polyomavirus VP1 complexes define a sialic acid binding site required for infection. PLoS Pathog.

[34] Bassaganas S., Perez-Garay M., Peracaula R. (2014). Cell surface sialic acid modulates extracellular matrix adhesion and migration in pancreatic adenocarcinoma cells. Pancreas.

[35] Meesmann H.M., Fehr E.M., Kierschke S., Herrmann M., Bilyy R., Heyder P., Blank N., Krienke S., Lorenz H.M., Schiller M. (2010). Decrease of sialic acid residues as an eat-me signal on the surface of apoptotic lymphocytes. J. Cell Sci..

[36] Sorensen A.L., Rumjantseva V., Nayeb-Hashemi S., Clausen H., Hartwig J.H., Wandall H.H., Hoffmeister K.M. (2009). Role of sialic acid for platelet life span: Exposure of beta-galactose results in the rapid clearance of platelets from the circulation by asialoglycoprotein receptor-expressing liver macrophages and hepatocytes. Blood.

[37] Kontou M., Weidemann W., Bork K., Horstkorte R. (2009). Beyond glycosylation: Sialic acid precursors act as signaling molecules and are involved in cellular control of differentiation of PC12 cells. Biol. Chem..

[38] Steirer L.M., Moe G.R. (2011). An antibody to de-*N*-acetyl sialic acid containing-polysialic acid identifies an intracellular antigen and induces apoptosis in human cancer cell lines. PLoS One.

